# Occurrence of the Retromolar Foramen in Dry Mandibles of South-Eastern Part of India: A Morphological Study with Review of the Literature

**DOI:** 10.1155/2014/296717

**Published:** 2014-09-29

**Authors:** Bhagath Kumar Potu, Vinod Kumar, Abdel-Halim Salem, Marwan Abu-Hijleh

**Affiliations:** ^1^Department of Anatomy, College of Medicine and Medical Sciences, Arabian Gulf University, 26671 Manama, Bahrain; ^2^Department of Anatomy, Sapthagiri Institute of Medical Sciences & Research Center, Bangalore 560010, India; ^3^Department of Anatomy, Faculty of Medicine, Suez Canal University, Ismailia 4111, Egypt

## Abstract

The retromolar foramen (RMF) is a rare anatomical structure situated in the retromolar fossa behind the third molar tooth. When it is present, the foramen is connected with the mandibular canal and is believed to transmit neurovascular structures that provide accessory source to the mandibular molars and the buccal area. Reports from the literature show that the presence of RMF could pose a challenge in complete blockage of the inferior alveolar nerve during mandibular surgeries. We report the incidence of retromolar foramen from ninety-four dry mandibles of south-eastern part of Karnataka State, India. The foramen was observed in 11 mandibles out of 94 included in the study (11.7%). In three mandibles, the foramen was present bilaterally (3.2%) and in three it was on the left side (3.2%) and in five it was on the right side (5.3%). For the first time, we also measured the dimensions of the retromolar area and distance of the foramen from third molar tooth to understand its risks during the surgical extraction of the lower third molar tooth. A thorough review of the literature has also been done to compare the present findings with the studies reported from the various populations.

## 1. Introduction

The RMF is an inconstant foramen situated in the central portion of the retromolar fossa which is bounded by the anterior border of ramus of the mandible and temporal crest. The foramen receives a canal of variable depth that normally arises from the mandibular canal behind the lower third molar, which is regarded as the retromolar canal (RMC) [1]. Normal morphological findings of the human mandible and its possible variations that occur have attracted special interest in the recent years in the field of odontostomatological surgeries [2]. One such anatomical variation which draws special attention in clinical dental practice is the RMF in the retromolar trigone (RMT). The trigone is bounded medially by temporal crest, laterally by the anterior border of the ramus, and anteriorly by the base of third molar tooth [3]. The RMF has generally been neglected in anatomical text books and this has been rarely studied or reviewed in the dental literature. The frequency of RMF has been reported in different populations showing a wide varying incidence between 3.2% and 72% [4–20]. However, there is no recent systematic study conducted on the RMF occurrence in the south-eastern part of Karnataka, India, with the morphometry we adopted; hence we report our findings on the occurrence of RMF in this population.

## 2. Material and Methods

In this study, all available 94 dry, fully ossified adult mandibles from the Department of Anatomy, Sapthagiri Institute of Medical Sciences and Research Center, Bangalore, south-eastern part of India, were included. In 94 dry mandibles, the presence of RMF was noted and the lengths of the anterior, medial, and lateral boundaries of the retromolar trigone were carefully measured using a digital vernier caliper. Wherever foramina were noticed, the distance of the RMF from the posterior border of socket for third molar, anterior border of the ramus, and lingula were also measured, respectively. All the measurements were performed independently by two individuals (Bhagath Kumar Potu and Vinod Kumar) for three times and averaged to arrive at accurate data. The mean, range, and standard deviation of all the measurements were statistically analyzed.

## 3. Results

**Figure 1:**
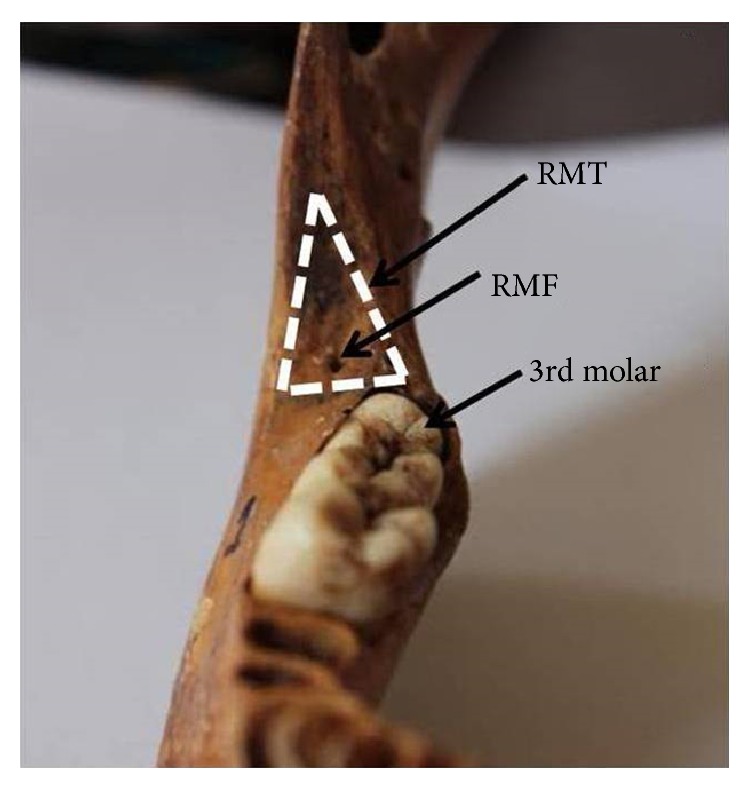
Showing the retromolar foramen (RMF) situated in the retromolar trigone (RMT) (dotted) behind the third molar tooth on right half of the mandible.

Retromolar foramen (RMF) was observed in 11 mandibles out of 94 included in the study (11.7%). In three mandibles, the foramen was observed bilaterally (3.2%) and in another three it was on the left side (3.2%) and in another five it was on the right side (5.3%). It was found that the length of medial boundary of the retromolar trigone varies between 19 and 30 mm, the lateral boundary varies between 21 and 32 mm, and the anterior boundary varies between 9 and 16 mm (Table 1). The RMF in our study was mostly located in medial aspect of the retromolar fossa close to lingula (Figure 1). The distances of the RMF from the posterior border of socket for third molar, anterior border of the ramus, and lingula are shown in Table 2. It was seen that the distance of RMF from third molar socket, from the anterior border of ramus, and also from the lingula varies between 4 and 11 mm, 3 and 11 mm, and 2 and 8 mm, respectively. The incidence of the RMF in our study is also compared with the studies conducted so far in different populations reported in the literature (Table 3).

## 4. Discussion

Considering the wide variation of incidence and the importance of this region in dental surgical procedures, the present study documented the prevalence of retromolar foramen in dry mandibles of a south-eastern Indian population sample. Percentage of the retromolar foramen occurrence in our study falls somewhere within the ranges reported from other Indian [9, 19], Turkish [20], Italian [18], Canadians of European descent [5], and Brazilian [11] populations (See Table 3). It has been observed that the neurovascular bundle of the RMF originated in the mandibular canal and penetrated into distal lamina dura of the distal root of the third molar [4]. Dentists in particular should be aware of this accessory innervation provided by RMF in the endodontic treatment [21]. Our findings show that the distance between the 3rd molar and RMF is being within the short range of 4–11 mm which is comparable with the recent reports published in the literature [8, 9, 19]. This close relation of RMF with 3rd molar could lead to damage of the structures traversing through RMF during the third molar extraction and could be a reason for postoperative hematomas as described by previous authors due to rupturing of the vessels in RMF [22–25]. The incidences reported from Indian population [8, 9, 19] are varying from 12 to 22%. The differences in the incidence of the RMF in these studies could be related to the differential origins of the Indian population. The presence of RMF with additional sensory nerve fibers was first reported by Sutton in 1974 [26]. He related the presence of this foramen to the failure of obtaining analgesia using classical anesthetic techniques [26]. Thus, the studies of the incidence of RMF are important in order to avoid failure in regional anesthetic techniques for blocking the inferior alveolar nerve and buccal nerve fibers [15, 27].

Singh reported that, during surgery of a third molar, he injured a nerve that crossed an unusual foramen located in the retromolar fossa [28]. After the surgery, it was found that the patient presented with paresthesia of the buccal mucosa from the retromolar region to the canine on the operated side. When this postoperative complication was investigated further, it was found that the nerve injured was a branch of the buccal nerve which crossed the RMF [28]. Anderson et al. [29] confirmed that the components in the RMF and canal are the nerves that provide innervation to the pulp of third molar, retromolar region and to the fibers of the temporalis and buccinator muscles. Thus damage to nerves of RMF and canal will disrupt the functions of temporalis and buccinator muscles. Pinsolle et al. [30] suggested that, because the RMF also allows the passage of vascular components, this may facilitate the spread of infection and metastases from the oropharynx to the blood circulation.

Keeping our measurements and observations in mind, one should be cautious when performing the following dental surgeries: third molar extraction, sagittal split osteotomies of the mandible, dieresis procedures, flap lifting, and placement of osseo-integrated implants [31].

## 5. Conclusion

This study reports the incidence of RMF in south-eastern part of Karnataka State, Indian sample, to form a basis for future better understanding of clinical and surgical practice related to the retromolar area. The RMF has great importance in the odontostomatological practice due to the prevalence of the pathological processes and complications related to the retromolar area involved in the practice. This could be the reason why this area is subject of a great number of surgical procedures. Knowledge of the location and contents of RMF should be carefully considered for choosing the best plan and consequently for optimizing anesthetic and surgery procedure during oral and maxillofacial procedures. We hope that this study would especially help surgeons in this part of the world to understand this area better. However, we declare that we had no availability of the radiological techniques for this present research investigations and this could be the limitation of our study. Our observations may alert the dental surgeons to conduct further studies and also to compare with studies of larger populations across the world to understand its origin and evolutionary importance.

## Figures and Tables

**Table 1:** Dimensions of the boundaries of retromolar trigone in the mandibles with presence of RMF.

Mandible number with RMF	Side	Borders of the retromolar trigone (mm)		
		Medial	Lateral	Anterior
9	Right	32	35	14
26	Left	35	35	16
32	Right	29	32	10
48	Left	46	41	12
51	Left	38	40	9
58	Right	43	46	11
58	Left	41	37	12
64	Right	42	38	15
72	Right	43	38	14
80	Right	34	35	12
88	Right	34	30	12
88	Left	30	32	13
91	Right	37	35	12
91	Left	36	35	10

Mean ± SD		**37.14 ± 5.23**	**36.36 ± 4.13**	**12.29 ± 1.98**

Min–Max		**29**–**46**	**30**–**46**	**9**–**16**

**Table 2:** Distance of RMF from 3rd molar socket, anterior border of the ramus, and lingula, respectively.

Mandible number	Side examined	Distance between the retromolar foramen and		
		3rd molar socket (mm)	Ant. border of ramus (mm)	Lingula (mm)
9	Right	4	5	8
26	Left	7	10	6
32	Right	8	3	6
48	Left	11	3	6
51	Left	6	4	2
58	Right	8	8	2
58	Left	4	5	3
64	Right	6	11	4
72	Right	4	9	2
80	Right	8	9	5
88	Right	5	8	6
88	Left	5	9	4
91	Right	6	4	3
91	Left	5	4	5

Mean ± SD		**6.21 ± 2.01**	**6.57 ± 2.82**	**4.43 ± 1.87**

Min–Max		**4**–**11**	**3**–**11**	**2**–**8**

**Table 3:** Incidence of retromolar foramen and canal in different populations reported in the literature.

Author	Year of study	Number of mandibles studied	Presence of retromolar foramen (%)	Population of the study
Schejtman et al. [4]	1967	18	13 (72)	Argentine aborigines
Ossenberg [5]	1987	86	7 (8.1)	Italian
		94	3 (3.2)	Japanese
		485	40 (8.2)	Eskimos
		11	1 (9.1)	Canadians of European descent
Sawyer and Kiely [6]	1991	234	18 (7.7)	American
Kodera and Hashimoto [15]	1995	41	8 (20)	Japanese
Pyle et al. [7]	1999	475	37 (7.8)	Caucasian (*n* = 226) Afro-American (*n* = 249)
Narayana et al. [8]	2002	242	53 (21.9)	Indian
Priya and Manjunath. [9]	2005	157	20 (12.7)	Indian
Lagrana et al. [16]	2006	50	9 (18)	Argentinean
Bilecenoglu and Tuncer [10]	2006	40	10 (25)	Turkish
Suazo et al. [11]	2008	294	38 (12.9)	Brazilian
von Arx et al. [12]	2011	121	31 (25.6)	Swiss
Kawai et al. [13]	2012	46	24 (52)	Japanese
Rossi et al. [14]	2012	222	59 (26.6)	Brazilian
Lizio et al. [18]	2013	233 (hemimandibles)	34 (14.6)	Italian
Athavale et al. [19]	2013	71	10 (14.1)	Indian
Orhan et al. [20]	2013	126	14 (11.1)	Turkish
Our study	2014	94	11 (11.7)	Indian
